# Let’s Connect: Impact Evaluation of an Intervention to Reduce Mental Health Disparities Among People Who are LGBTQ+ 

**DOI:** 10.1007/s10597-024-01231-4

**Published:** 2024-02-09

**Authors:** Shelley N. Facente, Xochitlquetzal Davila, Niko Kowell, Nicky Calma, Ming Ming Kwan, Shalika Gupta

**Affiliations:** 1 School of Public Health, University of California, Berkeley, 2121 Berkeley Way #5302, Berkeley, CA 94720 USA; 2 Facente Consulting, Richmond, CA USA; 3 San Francisco Community Health Center, San Francisco, CA USA

**Keywords:** Mental health, Transgender, Impact evaluation, Community intervention, LGBTQ

## Abstract

Lesbian, gay, bisexual, transgender, queer, or similarly identified (LGBTQ+) people experience substantial mental health disparities compared to heterosexuals. The “Let’s Connect” intervention was designed to improve mental health outcomes for LGBTQ+ people. This impact evaluation aimed to assess effectiveness of this intervention during its pilot phase, using a single arm pilot trial. Respondents completed baseline surveys at intervention start, a post survey on the last day of the intervention (at 6 weeks), then a follow-up survey 6 weeks after the intervention ended (at 12 weeks). Pre-post differences in outcomes were analyzed using paired t-tests, chi-square tests, and generalized estimating equations to evaluate impact on mental health outcomes at 6 and 12 weeks, and identify characteristics associated with loss to follow-up. The average value of all three outcome measures decreased substantially between the baseline and post surveys; all of these differences were highly statistically significant, and further decreased between the end of the intervention at 6 weeks and the 12 week follow-up survey. Let’s Connect participants did experience substantial improvements in mental health outcomes, on average, between the start and end of this intervention. Further study of this intervention using a randomized design and control group is warranted.

**Supplementary Information:**

The online version contains supplementary material available at 10.1007/s10597-024-01231-4.

## Introduction

People who are lesbian, gay, bisexual, transgender, queer, or similarly identified (LGBTQ+) often face unique stressors in life as a result of having a stigmatized sexual orientation or gender identity (Meyer, 2003; Suppes et al., 2021). As a result, they are more likely than heterosexual and cisgender^1^ people to experience anxiety, depression, and other psychological distress (Caldwell et al., 2023; Cochran & Mays, 2009; Salerno et al., 2023) or other indicators of poor mental health (Quinn & Earnshaw, 2011), including functional impairment (Sullivan et al., 2022) and/or feelings of isolation (Steinke et al., 2017). These mental health disparities are even more pronounced for LGBTQ+ people of color (Quinn et al., 2017; Robertson et al., 2021) and those who are trans and gender non-conforming (Carmel & Erickson-Schroth, 2016; Sarno et al., 2022).

A recent systematic review covering a 19 year period was able to locate only six individual-level mental health interventions tailored for LGBTQ+ people that had been systematically evaluated (Coulter et al., 2019). These included three clinical interventions (Costa et al., 2015; de Vries et al., 2014; Diamond et al., 2012) and three interventions that could be delivered in non-clinical settings, all focused exclusively on youth who were LGBTQ+ (Lucassen et al., 2015; Painter et al., 2018; Schwinn et al., 2015). None had a particular focus on LGBTQ+ people of color.

To fill this gap, we aimed to adapt and evaluate a new communications and mental health intervention for LGBTQ+ people that could be delivered in a non-clinical community-based setting, with a focus on people who are trans and gender non-conforming, and people of color. The original intervention, known as “Chai Chats,” was developed by Asian Women’s Shelter to support communication skill development for queer and trans women impacted by intimate partner violence. After an extremely positive reception within the local community (Ming Ming Kwan, personal communication), we wondered whether it could be adapted to focus on all LGBTQ+ people more broadly, and expand the intimate partnership focus to strengthening supportive networks overall, thus potentially improving mental health outcomes (McConnell et al., 2015; Terry, 2021). Three staff members who identified as LGBTQ+ and had experience with teaching and curriculum design adapted the intervention goals and focus for LGBTQ+ people more broadly, with the advice of two mental health professionals with expertise serving LGBTQ+ populations of color. This adapted version of Let’s Connect was then piloted and evaluated as part of the California Reducing Disparities Project (CRDP), a 5 year initiative of the California Department of Public Health Office of Health Equity.

Our evaluation was designed to use validated measures of psychological distress, functional impairment, and sense of isolation to answer two research questions: (1) What is the association between participating in the Let’s Connect intervention and mental health outcomes for LGBTQ+ people, at both 6 weeks (from the beginning of the Let’s Connect intervention until the end of the program), and 12 weeks (after six weeks post-intervention)? (2) What characteristics are associated with program attrition for Let’s Connect participants?

## Methods

### Intervention Description

Let’s Connect is a program with a standard curriculum based on eight two-hour sessions designed to improve communication and coping skills (to reduce psychological distress and improve function), recognize strengths and areas of growth in both self and one’s support network (to reduce sense of social isolation), and appreciate the challenges related to intersections of gender, sexual orientation, race, class, and age. Due to the COVID-19 pandemic, the intervention was delivered through an all-virtual, 6 week format with 2-hour zoom sessions each week. The curriculum was led by two LGBTQ+ facilitators, one of whom was a mental health provider. The inclusion of a mental health provider in the facilitation team allowed for early identification and linkage of participants with mental health risk factors or unmet mental health needs, and immediate crisis intervention when necessary.

### Recruitment and Enrollment

Participants were recruited online via electronic flyers shared through social media and other professional and personal networks. Participants were asked to complete the baseline survey prior to or during the start of the first session, then a post survey on the last day of the intervention (at 6 weeks), and a follow-up survey at 12 weeks. Any person aged 18 or over with a California residence zip code who self-identified as LGBTQ+ and was willing to provide written informed consent and commit to the entire Let’s Connect intervention was eligible to enroll. Participants were not required to report psychological distress as a requirement for entry into the intervention, as this intervention was designed as a group-specific intervention for both early intervention and primary prevention of mental health disorders.

Once individuals indicated interest in participating they were directed to an online summary of the intervention and evaluation study, along with a link to a secure DocuSign portal to review and sign the informed consent form. Participants were not randomized to the Let’s Connect intervention for ethical reasons: in many cases the services available as part of these intervention components were the only culturally competent options available to participants. Since no randomization took place and there was no control group, our statistical models controlled for baseline health and demographic characteristics, but causality of the intervention’s impact on mental health outcomes could not be inferred.

### Data Collection

The main mechanism for data collection in this study was a series of online surveys administered in English or in Spanish. Surveys were created using Questionnaire Development System [Nova Research Company, Silver Spring, MD] and were taken by participants via a secure web portal. The baseline survey contained questions about demographics, healthcare access history, and mental health status (see *Outcome Measures)* built upon measures from the California Health Interview Survey (UCLA Center for Health Policy Research, 2021), which have been validated in both English and Spanish. The post and follow-up surveys were identical and included all baseline mental health status questions. Participants received a $10 payment for completing the baseline survey at the first session, plus another $10 for each Let's Connect session they attended (including the final session where the post survey was completed), and a $100 payment for completing the follow-up survey 12 weeks after intervention end.

### Research Questions and Analysis Plan

Research Question 1 assessed changes in mental health outcomes before and after participation in Let’s Connect with varying periods of follow-up. Therefore, associations were estimated with population average models using generalized estimating equations (GEE) for differences in mental health outcome scores (as described in the next section) from baseline to each post and follow-up survey for participants with complete survey series, with time as an independent categorical variable. Here, complete survey series means that the individual participated in the baseline, post, and follow-up surveys. All available covariate and outcome data were used to estimate the GEE models; within this group, there was some missingness in covariate and outcome data. The parameter estimates from the GEE models represent the mean change in mental health outcome scores between the beginning and end of the intervention, and the mean change in the mental health outcome scores between the beginning of the intervention and the end of the 6 week follow-up period, to assess durability of effect. We used an independent working correlation model with robust standard errors and an α of 0.05 for significance testing.

Research Question 2 was measured by comparing covariates of participants at baseline who did not complete any further surveys with those who completed the survey series. We used paired t-tests to assess significance of mean differences in continuous baseline values for the three outcome variables as well as age, gender affirmation by others, and reported experiences of stigma or discrimination, between those who completed the survey series and those lost to follow-up after baseline. We similarly conducted a chi-square test for independence to assess differences in baseline values for categorical covariates, between those who completed the survey series and those lost to follow-up. For all statistical tests used to answer this research question, we used α of 0.05.

#### Outcome Measures

Data were collected to measure three mental health outcomes, which were assessed using the same questions at all three surveys. First, we used a validated measure for psychological distress, the Kessler 6 (Kessler et al., 2003), which measures an individual’s psychological distress (e.g., feelings of nervousness and hopelessness) over the previous 30 day period; this score ranges from 0 to 24, with a higher score indicating more distress. To calculate a participant’s Kessler 6 score, six survey questions were coded from 0 (“none of the time”) to 4 (“all of the time”) and the sum was taken over all six items. For individuals missing no more than half of these distress-related survey questions, missing values to one or more questions were imputed using the individual’s mean, calculated by taking the mean over the non-missing items (Shrive et al., 2006; Siddiqui, 2015). Participants missing more than half of the Kessler 6 items were excluded from the analysis.

Second, we used a four-item version of the Sheehan Disability Scale, a validated measure for functioning (Sheehan, 2008), adapted by the CHIS to evaluate whether an individual’s emotions interfered with work performance, household chores, social life, or relationships with friends and family in the previous month. This scale ranges from 0 to 8, with a higher score indicating more impaired functioning. Each of the four relevant survey questions were coded from 0 (“not at all”) to 2 (“a lot”), and the sum was taken over the four items. For individuals missing responses to no more than half of the functioning questions, missing values were again imputed using the individual mean.

Third, we used a composite of two survey questions (referred to as the “isolation” outcome in our results: “About how often during the past 30 days were you made to feel unimportant, or like your thoughts, feelings, or opinions don’t matter in society?” and “About how often during the past 30 days were you feeling alone, separated from, cut off from the world beyond your family, school, and friends?”) for which responses were on a 5-point Likert scale from “None of the time” (0) to “All of the time” (4). Responses to these questions were summed. If participants only responded to one of these two questions, the response from the completed question was used to impute the missing value. Participants missing responses to both questions were excluded from this portion of the analysis.

#### Baseline Covariates

Additional demographic and behavioral information used as covariates in our statistical models was collected on the baseline survey (Table 1). Race/ethnicity, housing status, gender affirmation, gender conformity, sexual orientation, and age were used as covariates in the multivariate models for Research Question 1, selected based on a directed acyclic graph developed by the research team.

**Table 1:** Baseline covariates and method of summary

Covariate	Method of classification
Age	Count (age rounded to nearest whole number)
Race/ethnicity	White (self-reported white ethnicity with no other race/ethnic categories selected); BIPOC (self-reported monorace of Black, Latinx, Asian, Pacific Islander, Native American, Other); Multiracial (self-selection of more than one racial/ethnic category)
Housing status	Stably housed (rent/own); Unstably housed/Unhoused (e.g., shelter, couch surfing, living on the streets or in a park). Unstably housed and unhoused were collapsed into one category for analysis due to very small numbers of unhoused people after the intervention became virtual starting May of 2020
Sexual orientation	Gay/lesbian; bisexual; other. For purposes of analysis, people who identified as heterosexual were grouped into the “other” category due to small numbers, as this was an LGBTQ-focused intervention. People who identified as “queer” were categorized according to other choices (i.e., “gay” and “queer” would result in a categorization as “gay”); if no other options were picked they were categorized as “other.”
Gender identity	Cisgender (man only, or woman only, and gender aligned with sex at birth); or Transgender/Other (all other gender/sex combinations). Small sample size prevented further breakdown of the transgender/other gender category in our statistical models
Gender conformity	Gender normative (if gender = man and both self-perception and others’ perception of appearance, style and mannerisms is “somewhat” to “very” masculine; alternatively, if gender = woman and both self-perception and others’ perception of appearance, style and mannerisms is “somewhat” to “very” feminine); gender non-conforming (all other combinations)
Gender affirmation	Mean score of 11 questions related to level of gender affirmation (from 0 = totally invalidating to 4 = totally affirming) for various relationships, including parents/guardians, siblings, extended family, children, friends, partner(s), coworkers, neighbors, medical providers, mental health providers, and an additional "Other" group that individuals could specify themselves
Experience of stigma/discrimination	Mean score on 9 questions asking whether subject has experienced different forms of stigma or discrimination, on a scale of 0 (never) to 5 (almost every day)
Health insurance status	Currently insured with mental health coverage; currently insured but without mental health coverage; insured within the past 12 months but not currently insured; or uninsured now and for the past 12 months
Health-seeking behavior	Did not seek help but did not think they needed any; thought they needed help but did not seek any; sought help. Participants were classified based on their responses to a series of questions regarding if they felt they needed to seek help from professionals (including but not limited to culturally-based healers, religious/spiritual leaders, health workers, promoters/*promotoras*, peer counselors, case managers, physicians or general practitioners, and/or mental health professionals) and whether or not the individuals met with any of these types of professionals in the past 12 months
Location	In Bay Area (zip code corresponding to San Francisco, Alameda, Contra Costa, Marin, San Mateo, or Santa Clara counties); Outside of Bay Area (zip code corresponding to another California county)

### Research Ethics

As an evaluation of the success of an established intervention in achieving its objectives in a specific population, and in which the information gained will be used to make improvements in the program, this evaluation was determined to be Exempt from the State of California’s Committee for the Protection of Human Subjects (CPHS) and did not require approval by CPHS. Nonetheless, all participants signed a written form giving informed consent for their data to be used in the research.

## Results

There were eight cycles of Let’s Connect conducted from May 2020 through June 2021. From these eight cycles there was a final sample size of 116 people who took the baseline survey and were therefore included in the analysis of Research Question 2, 71 (61%) of whom completed the survey series with sufficient completeness in the outcome measures to be included in the analysis of Research Question 1. Details on the demographics of individuals retained in the final sample is available in Table 2 and more detail about the timing and attendance of each of the eight cycles can be found in Supplemental Table S1. For Table 2 and for analyses related to Research Question 2, we excluded 3 people who took a baseline and follow-up survey but skipped the post survey, and 3 who took a baseline and post survey but were lost to follow-up before the follow-up survey.

**Table 2:** Descriptive statistics of key demographics in the final sample

	Participants who completed the survey series (baseline, post, and follow-up surveys)	Participants who were lost to follow-up (completed baseline only; no post or follow-up survey)	Overall
	(n = 71)	(n = 45)	(n = 116)
Race/ethnicity			
American Indian	0 (0%)	0 (0%)	0 (0%)
Black	1 (1.4%)	1 (2.2%)	2 (1.7%)
Latinx	8 (11.3%)	6 (13.3%)	14 (12.1%)
Asian	21 (29.6%)	4 (8.9%)	25 (21.6%)
Native Hawaiian	0 (0%)	0 (0%)	0 (0%)
White	13 (18.3%)	15 (33.3%)	28 (24.1%)
Other	0 (0%)	0 (0%)	0 (0%)
Multiracial	25 (35.2%)	18 (40.0%)	43 (37.1%)
Missing	3 (4.2%)	1 (2.2%)	4 (3.4%)
Gender identity			
Cisgender	19 (26.8%)	10 (22.2%)	29 (25.0%)
Transgender/Other	52 (73.2%)	34 (75.6%)	86 (74.1%)
Missing	0 (0%)	1 (2.2%)	1 (0.9%)
Gender conformity			
Normative	16 (22.5%)	11 (24.4%)	27 (23.3%)
Nonconforming	54 (76.1%)	33 (73.3%)	87 (75.0%)
Missing	1 (1.4%)	1 (2.2%)	2 (1.7%)
Gender affirmation by others			
Mean (SD)	2.94 (0.821)	2.98 (0.797)	2.95 (0.809)
Median [minimum, maximum]	3.00 [1.17, 4.00]	3.00 [0.70, 4.00]	3.00 [0.70, 4.00]
Age (years)			
Mean (SD)	28.9 (8.67)	36.3 (14.2)	31.8 (11.7)
Median [minimum, maximum]	26.3 [19.4, 61.6]	31.5 [18.4, 70.6]	28.3 [18.4, 70.6]
Housing status			
Stably housed	56 (78.9%)	35 (77.8%)	91 (78.4%)
Unstably housed/unhoused	15 (21.1%)	8 (17.8%)	23 (19.8%)
Missing	0 (0%)	2 (4.4%)	2 (1.7%)
Insurance status			
Insured with mental health coverage	47 (66.2%)	24 (53.3%)	71 (61.2%)
Insured but no mental health coverage	14 (19.7%)	11 (24.4%)	25 (21.6%)
Insured in past 12 months but not currently	6 (8.5%)	5 (11.1%)	11 (9.5%)
Uninsured now and for past 12 months	4 (5.6%)	1 (2.2%)	5 (4.3%)
Health seeking behavior			
Did not need or use services	3 (4.2%)	3 (6.7%)	6 (5.2%)
Needed services but did not go	7 (9.9%)	8 (17.8%)	15 (12.9%)
Used services	60 (84.5%)	30 (66.7%)	90 (77.6%)
Missing	1 (1.4%)	4 (8.9%)	5 (4.3%)
Sexual orientation			
Heterosexual	1 (1.4%)	2 (4.4%)	3 (2.6%)
Gay/Lesbian	4 (5.6%)	10 (22.2%)	14 (12.1%)
Bisexual	19 (26.8%)	12 (26.7%)	31 (26.7%)
Other	47 (66.2%)	20 (44.4%)	67 (57.8%)
Missing	0 (0%)	1 (2.2%)	1 (0.9%)
Reported experience of stigma/discrimination			
Mean (SD)	1.99 (1.06)	2.23 (1.24)	2.08 (1.14)
Median [minimum, maximum]	1.89 [0, 4.78]	1.89 [0, 5.00]	1.89 [0, 5.00]
Location			
Outside of bay area (in California)	25 (35.2%)	11 (24.4%)	36 (31.0%)
In bay area	45 (63.4%)	31 (68.9%)	76 (65.5%)
Missing	1 (1.4%)	3 (6.7%)	4 (3.4%)

*SD* standard deviation

### Research Question 1: Association Between Intervention Participation and Mental Health

The average value of all three outcome measures decreased between the baseline and post surveys (Tables 3 and 4); all of these differences were highly statistically significant (p < 0.01). Those same decreases were maintained—and in fact further decreased—between the baseline and the 12 week follow-up survey (again p < 0.01). However, the decreases in mean outcome scores from post to follow-up surveys were not statistically significant. Between the baseline and post surveys, the mean Kessler 6 score decreased by an estimated 2.4 points on the scale of 0–24, when adjusting for race/ethnicity, housing status, gender affirmation, gender conformity, sexual orientation, and age, and this decrease was statistically significant (p < 0.01) (Table 5). Similarly, between the baseline and follow-up surveys, the mean Kessler 6 score decreased by an estimated 3.03 points, adjusting for the same covariates, and again this decrease was statistically significant (p < 0.01). Throughout the study, the mean Kessler 6 score was 5.49 points higher among those who were unstably housed or unhoused as compared to those living in stable housing after adjusting for time and the other covariates, and this difference was statistically significant (p < 0.01). None of the other covariates showed statistically significant differences related to the Kessler 6 scores during the study.

**Table 3:** Means of outcome measures at baseline, post and follow-up surveys for those with complete series (n = 71)

	Baseline survey			Post survey			Follow-up survey		
	n	Mean	SD	n	Mean	SD	n	Mean	SD
Kessler 6	71	13.48	5.65	71	11.11	5.8	71	10.39	5.7
Sheehan disability scale	70	6.41	2.0	70	4.76	2.36	71	4.65	2.36
Isolation	71	4.83	1.99	71	3.99	2.15	71	3.61	2.22

*SD* standard deviation

**Table 4:** Unadjusted differences in outcome measures for all participants who completed the survey series and had non-missing outcome values at relevant timepoints, using paired t-tests

	Change from baseline to post			Change from post to follow-up			Change from baseline to follow-up		
Outcome measure	n	Estimate (95% CI)	p-value	n	Estimate (95% CI)	p-value	n	Estimate (95% CI)	p-value
Kessler 6	71	− 2.37 (− 3.35, − 1.39)	< 0.01	71	− 0.72 (− 1.54, 0.10)	0.08	71	− 3.08 (− 4.06, − 2.11)	< 0.01
Sheehan disability scale	70	− 1.57 (− 2.08, − 1.07)	< 0.01	70	− 0.1 (− 0.62, 0.41)	0.69	71	− 1.70 (− 2.28, − 1.12)	< 0.01
Isolation	71	− 0.85 (− 1.40, − 0.29)	< 0.01	71	− 0.38 (− 0.82, 0.06)	0.09	71	− 1.23 (− 1.77, − 0.69)	< 0.01

*95% CI*  95% confidence interval

**Table 5:** Adjusted estimates for differences in outcomes for individuals with complete survey series, using generalized estimating equations and an independent working correlation structure

	Kessler 6	Sheehan Disability Scale	Isolation
	Estimate (95% CI)	Estimate (95% CI)	Estimate (95% CI)
Post	− 2.40*** (− 3.38, − 1.42)	− 1.74*** (− 2.23, − 1.25)	− 0.94*** (− 1.49, − 0.40)
Follow-up	− 3.03*** (− 4.03, − 2.03)	− 1.88*** (− 2.45, − 1.30)	− 1.24*** (− 1.78, − 0.70)
Race/ethnicity			
BIPOC	1.28 (− 1.63, 4.19)	− 0.29 (− 1.42, 0.84)	0.64 (− 0.29, 1.57)
Multiracial	0.88 (− 2.35, 4.10)	− 0.25 (− 1.47, 0.97)	0.74 (− 0.18, 1.66)
White	Ref	Ref	Ref
Housing status			
Unstably housed/unhoused	5.49*** (3.01, 7.96)	0.94** (0.07, 1.80)	1.45*** (0.66, 2.24)
Stably housed	Ref	Ref	Ref
Gender affirmation	− 1.44* (− 3.02, 0.15)	− 0.42 (− 0.99, 0.15)	− 0.62*** (− 1.09, − 0.16)
Gender conformity			
Gender nonconforming	− 1.2 (− 4.29, 1.89)	0.12 (− 1.02, 1.26)	− 0.53 (− 1.42, 0.36)
Gender normative	Ref	Ref	Ref
Sexual orientation			
Bisexual	3.77* (− 0.23, 7.76)	2.53*** (1.42, 3.64)	1.99*** (0.97, 3.02)
Other orientation	2.15 (− 1.46, 5.76)	2.26*** (1.21, 3.31)	1.30*** (0.42, 2.18)
Gay/lesbian	Ref	Ref	Ref
Age	− 0.11 (− 0.25, 0.03)	− 0.04 (− 0.11, 0.03)	0.01 (− 0.02, 0.05)
Observations	204^a^	202^b^	204^a^

*95% CI*  95% confidence interval, *BIPOC*  black, indigenous, and other people of color

*p < 0.1; **p < 0.05; ***p < 0.01

^a^For these measures, there were 68 unique people with 1 observation at each of the three timepoints

^b^For this measure, there were 66 unique people with 1 observation at each of 3 timepoints, and 2 people with 1 observation at 2 of the 3 timepoints

For the Sheehan Disability Scale, the score between the baseline and post surveys decreased by an estimated 1.74 points on the scale of 0–8, when adjusting for the same covariates, and this decrease was statistically significant (p < 0.01). Similarly, between the baseline and follow-up surveys, the adjusted mean Sheehan Disability Scale score decreased by an estimated 1.88 points, and this decrease was again statistically significant (p < 0.01). Like with the Kessler 6, throughout the study the mean Sheehan Disability Scale score was 0.94 points higher among those who were unstably housed or unhoused as compared to those living in stable housing after adjusting for time and the remaining covariates (p < 0.05). However, with the Sheehan Disability Scale, the mean scores throughout the study were 2.53 points higher among those who identified as bisexual (p < 0.01) and 2.26 points higher among those who identified with another sexual orientation (p < 0.01), when compared to those who identified as gay/lesbian, after adjusting for time and the other covariates. The Sheehan Disability Scale evaluates whether an individual’s emotions are interfering with work performance, household chores, social life, or relationships with friends and family; this statistically significant difference in function by sexual orientation is a notable finding that warrants further exploration. None of the other covariates showed significant differences related to the Sheehan Disability Scale scores.

Finally, between the baseline and post surveys, the mean isolation score decreased by an estimated 0.94 points on the scale of 0–8, adjusting for the same covariates, and this decrease was statistically significant (p < 0.01). Between the baseline and follow-up surveys, the adjusted mean isolation score decreased by an estimated 1.24 points, and this decrease was again statistically significant (p < 0.01). Like with the other outcome measures, throughout the study the mean isolation score was 1.45 points higher among those who were unstably housed or unhoused as compared to those living in stable housing, after adjusting for time and the other covariates (p < 0.01). Like with the Sheehan Disability Scale, the mean isolation scores throughout the study were 1.99 points higher among those who identified as bisexual (p < 0.01) and 1.30 points higher among those who identified with another sexual orientation (p < 0.01), when compared to those who identified as gay/lesbian, after adjusting for time and the other covariates. Again, this indicates worse mental health outcomes overall for those who are bisexual or have another sexual orientation compared to those who identify as gay or lesbian. Finally, for every one-unit increase in the composite score for gender affirmation by others reported by participants (on a scale of 0–4), there was a statistically significant reduction in mean isolation score throughout the study (− 0.62 points lower, 95% CI − 1.09, − 0.16)—unsurprising, as being surrounded by friends, family, and coworkers who affirm one’s gender could reasonably be expected to reduce one’s sense of isolation and exclusion.

For all three outcome measures, the mean score at the follow-up survey was slightly less than at the post survey (Table 3). To further explore durability of effect, we calculated the Pearson correlation coefficient for each of the participants with complete survey series for each outcome score. The correlation of baseline and post outcome measures ranged from relatively low (0.37) for isolation scores to strong (0.69 and 0.74) for the Sheehan Disability Scale and Kessler 6, respectively. The correlation of post and follow-up outcome measures ranged from moderate (0.58 and 0.63 for the Sheehan Disability Scale and isolation score, respectively) to strong (0.82) for the Kessler 6. Finally, the correlation of baseline and follow-up outcome measures ranged from relatively low (0.36 and 0.42 for the Sheehan Disability Scale and isolation score, respectively) to strong (0.74) for the Kessler 6.

### Research Question 2: Characteristics Associated with Loss to Follow-Up

The mean Kessler 6 and isolation scores for people who were lost to follow-up after the baseline survey were slightly higher than those with observations in baseline, post, and follow-up surveys, though the mean Sheehan Disability Scale scores were identical between the two groups (Table 6). Ultimately, there were no significant differences between the group lost to follow up after baseline and the group who completed the survey series with respect to race/ethnicity, housing status, gender identity, gender conformity, gender affirmation by others, insurance status, health-seeking behavior, location (living in the Bay Area), reported experiences of stigma or discrimination, Kessler 6 score, Sheehan Disability Scale, or isolation score at the baseline survey. However, there were significant differences with respect to sexual orientation between the two groups (p = 0.02), with a greater number of people who completed the survey series having bisexual or “other” sexual orientation (as compared to gay/lesbian or heterosexual orientation) than those lost to follow-up. Similarly, there were statistically significant differences for age (p = 0.003), with those who completed the survey series being considerably younger, on average, than those who were lost to follow-up.

**Table 6:** Mean of outcome measures at baseline, comparing those who completed the series to those who were lost to follow-up after the baseline survey

	Overall (n = 116)			Participants who completed baseline, post, and follow-up surveys (n = 71)			Participants who completed baseline survey only (lost to follow-up) (n = 45)		
	n	Mean	SD	n	Mean	SD	n	Mean	SD
Kessler 6	115	13.78	5.63	71	13.48	5.65	44	14.27	5.62
Sheehan disability scale	111	6.41	1.96	70	6.41	2	41	6.41	1.9
Isolation	113	4.87	2.02	71	4.83	1.99	42	4.93	2.08

*SD* standard deviation

## Discussion

In this impact evaluation we found that the Let’s Connect intervention was significantly effective at improving measures of psychological distress, functioning, and isolation for LGBTQ+ participants during the course of the intervention, with effects continuing to deepen 6 weeks after the intervention ended. This uplifts Let’s Connect as a potential model to replicate elsewhere, as few interventions developed to improve mental health among LGBTQ+ people have been evaluated, especially for those who are no longer youth or young adults, or those designed to address the intersections of gender, sexual orientation, race, and class. People with overlapping stigmatized identities (e.g., BIPOC LGBTQ+ people) often suffer even worse health disparities (Quinn & Dickson-Gomez, 2016; Quinn et al., 2017; Robertson et al., 2021), and thus intersectionality was an underlying theme of the Let’s Connect intervention by design, and our results suggest this was an important feature.

Mental health interventions specific to LGBTQ+ people are sorely needed, as LGBTQ+ people continue to have persistent risk factors for suicide (Transgender Law Center, 2012), substance use (Batchelder et al., 2021; Diaz et al., 2021; Klare et al., 2020), and poor health outcomes for HIV (Dorcé-Medard et al., 2021), cancer (Fuchs et al., 2021; Junejo & Sheikh, 2021), and hepatitis C (Deacon et al., 2013; Wang et al., 2021). This is especially true for those on the fringes of the LGBTQ+ community, such as those with less mainstream identities (such as being bisexual or having another less-well-recognized sexual orientation, or being trans or gender non-conforming), and those who were unhoused or unstably housed (Batchelder et al., 2021). This aligns with our study findings, as participants who were unstably housed/unhoused and those who identified as bisexual or another sexual orientation regularly had poorer mental health outcomes than their stably housed or gay/lesbian-identified counterparts.

## Limitations

This evaluation had a number of limitations. First, we were not able to evaluate the Let’s Connect intervention using a randomized trial design or control group. While our analysis does indicate that Let’s Connect affected mental health-related risk and protective factors over time, those who chose to participate in Let’s Connect are likely to be different from those who did not, according to factors we did not measure and control for in this analysis, and we are limited in our ability to attribute changes in mental health outcomes to the intervention itself. Second, individuals with relatively low or high scores on any of the outcome measures at baseline may be less likely to have such extreme values at follow-up, which may not be indicative of a true change in outcomes but rather “regression to the mean”—this effect could not be accounted for with our pre-post analysis study design. Third, we did not adjust for multiple comparisons in this analysis, increasing the chance that some of the statistically significant findings were significant by chance, and not representative of a true effect. Finally, because of the way that recruitment for the study was conducted (i.e. online advertisements through personal and professional networks), it is likely that the evaluation sample is not representative of the large population of eligible LGBTQ+ Californians, nor LGBTQ+ people nationally.

## Conclusion

Ultimately, Let’s Connect participants did experience substantial improvements in mental health outcomes, on average, between the start and end of this intervention; those improvements in psychological distress, function, and isolation continued and even deepened in the 6 weeks following the end of the intervention cycle. Our study design prevents a causal interpretation of these results; however, further study of this intervention using a randomized design is warranted given the positive associations between intervention participation and mental health outcomes in this single arm pilot trial.

### Supplementary Information

Below is the link to the electronic supplementary material.

Supplementary file1 (DOCX 14 kb)

## Footnotes

1 Cisgender people are those whose gender identity and sex assigned at birth are concordant (i.e., not transgender).
