# Supplementary material for: Let’s Connect: Impact Evaluation of an Intervention to Reduce Mental Health Disparities Among People Who are LGBTQ+
Source: Community Ment Health J. 2024 Feb 9;60(4):754–63. doi: 10.1007/s10597-024-01231-4 (PMC11001695; doi:10.1007/s10597-024-01231-4)
Supplement: Supplementary file 1 — Supplementary file1 (DOCX 14 kb) [file 10597_2024_1231_MOESM1_ESM.docx]

**Table S1. Let’s Connect cohort cycles from May 2020 through April 2021, with details of cycle start date and participation.**

| **Cohort start date** | **# started cycle and took baseline survey** | **# completed cycle and took post survey** | **# completed follow-up survey** | **% retention from baseline through follow-up** |
| --- | --- | --- | --- | --- |
| May 2020 | 8 | 5 | 5 | 63% |
| Sept 2020 | 17 | 13 | 13 | 76% |
| Oct 2020 | 17 | 11 | 10^†^ | 59% |
| Dec 2020 | 17 | 12 | 12 | 71% |
| Feb 2021 (#1)* | 23 | 18 | 18 | 78% |
| Feb 2021 (#2)* | 16 | 6 | 7^†^ | 44% |
| Apr 2021 (#1)* | 23 | 7 | 6 | 26% |
| Apr 2021 (#2)* | 4 | 1 | 2^†^ | 50% |

* In February and April 2021, two cycles were run concurrently.

^†^Note that some of the people who completed “post” surveys did not complete “mid” surveys, and therefore were not considered to have completed the series.
